# Leucine-rich alpha-2 glycoprotein is a potential biomarker to monitor disease activity in inflammatory bowel disease receiving adalimumab: PLANET study

**DOI:** 10.1007/s00535-021-01793-0

**Published:** 2021-05-03

**Authors:** Shinichiro Shinzaki, Katsuyoshi Matsuoka, Hiroki Tanaka, Fuminao Takeshima, Shingo Kato, Takehiro Torisu, Yuki Ohta, Kenji Watanabe, Shiro Nakamura, Naoki Yoshimura, Taku Kobayashi, Akiko Shiotani, Fumihito Hirai, Sakiko Hiraoka, Mamoru Watanabe, Minoru Matsuura, Shohei Nishimoto, Shinta Mizuno, Hideki Iijima, Tetsuo Takehara, Tetsuji Naka, Takanori Kanai, Takayuki Matsumoto

**Affiliations:** 1 Department of Gastroenterology and Hepatology, Osaka University Graduate School of Medicine, 2-2 Yamadaoka, Suita, Osaka 565-0871 Japan; 2 Inflammatory Bowel Disease Center, Toho University Sakura Medical Center, Chiba, Japan; 3 IBD Center, Sapporo Kosei General Hospital, Hokkaido, Japan; 4 Department of Gastroenterology and Hepatology, Nagasaki University Graduate School of Biomedical Sciences, Nagasaki, Japan; 5 Department of Gastroenterology and Hepatology, Saitama Medical Center, Saitama Medical University, Saitama, Japan; 6 Department of Medicine and Clinical Science, Graduate School of Medical Sciences, Kyushu University, Fukuoka, Japan; 7 Department of Gastroenterology, Graduate School of Medicine, Chiba University, Chiba, Japan; 8 Division of Internal Medicine, Center for Inflammatory Bowel Disease, Hyogo College of Medicine, Hyogo, Japan; 9 Second Department of Internal Medicine, Osaka Medical College, Osaka, Japan; 10 Department of Internal Medicine, Division of IBD, Tokyo Yamate Medical Center, Tokyo, Japan; 11 Center for Advanced IBD Research and Treatment, Kitasato University Kitasato Institute Hospital, Tokyo, Japan; 12 Division of Gastroenterology, Department of Internal Medicine, Kawasaki Medical School, Okayama, Japan; 13 Department of Gastroenterology, Fukuoka University Faculty of Medicine, Fukuoka, Japan; 14 Department of Gastroenterology and Hepatology, Okayama University Graduate School of Medicine, Dentistry and Pharmaceutical Sciences, Okayama, Japan; 15 Department of Gastroenterology and Hepatology, Advanced Research Institute, Tokyo Medical and Dental University, Tokyo, Japan; 16 Department of Gastroenterology and Hepatology, Kyorin University School of Medicine, Tokyo, Japan; 17 Eisai Co. Ltd, Tokyo, Japan; 18 Division of Gastroenterology and Hepatology, Department of Internal Medicine, Keio University School of Medicine, Tokyo, Japan; 19 Center for Intractable Immune Disease, Kochi Medical School, Kochi University, Kochi, Japan; 20 Division of Gastroenterology, Department of Medicine, Iwate Medical University, Iwate, Japan

**Keywords:** Crohn’s disease, Ulcerative colitis, Leucine-rich alpha-2 glycoprotein, Biomarker

## Abstract

**Background:**

This multicenter prospective study (UMIN000019958) aimed to evaluate the usefulness of serum leucin-rich alpha-2 glycoprotein (LRG) levels in monitoring disease activity in inflammatory bowel disease (IBD).

**Methods:**

Patients with moderate-to-severe IBD initiated on adalimumab therapy were enrolled herein. Serum LRG, C-reactive protein (CRP), and fecal calprotectin (fCal) levels were measured at week 0, 12, 24, and 52. Colonoscopy was performed at week 0, 12, and 52 for ulcerative colitis (UC), and at week 0, 24, and 52 for Crohn’s disease (CD). Endoscopic activity was assessed using the Simple Endoscopic Score for Crohn’s Disease (SES-CD) for CD and the Mayo endoscopic subscore (MES) for UC.

**Results:**

A total of 81 patients was enrolled. Serum LRG levels decreased along with improvements in clinical and endoscopic outcomes upon adalimumab treatment (27.4 ± 12.6 μg/ml at week 0, 15.5 ± 7.7 μg/ml at week 12, 15.7 ± 9.6 μg/ml at week 24, and 14.5 ± 6.8 μg/ml at week 52), being correlated with endoscopic activity at each time point (SES-CD: *r* = 0.391 at week 0, *r* = 0.563 at week 24, *r* = 0.697 at week 52; MES: *r* = 0.534 at week 0, *r* = 0.429 at week 12, *r* = 0.335 at week 52). Endoscopic activity better correlated with LRG compared to CRP and fCal on pooled analysis at all time points (SES-CD: LRG: *r* = 0.636, CRP: *r* = 0.402, fCal: *r* = 0.435; MES: LRG: *r* = 0.568, CRP: 0.389, fCal: *r* = 0.426).

**Conclusions:**

Serum LRG is a useful biomarker of endoscopic activity both in CD and UC during the adalimumab treatment.

**Supplementary Information:**

The online version contains supplementary material available at 10.1007/s00535-021-01793-0.

## Introduction

The etiology of inflammatory bowel disease (IBD), such as Crohn’s disease (CD) and ulcerative colitis (UC), is still unknown, and the number of patients with these diseases is increasing worldwide [1, 2]. Recent therapeutic progress has enabled us to select several therapeutic options for IBD, including anti-tumor necrosis factor (TNF) agents and immunosuppressants [3]. Using these medications in appropriate situations, the goal of the treatment has changed from clinical remission to mucosal healing, which has decreased the risks for hospitalization and surgical intervention.

The treat-to-target strategy requires frequent monitoring of disease activity. Recently updated STRIDE-II statements have mentioned that the most important long-term achievable treatment targets for IBD patients are clinical remission, endoscopic healing, restoration of quality of life, and absence of disability [4]. Colonoscopy is also the current gold standard for monitoring mucosal healing in IBD patients [5]. However, frequent endoscopy is difficult to perform, because it is time-consuming, invasive, and labor-intensive. Serum C-reactive protein (CRP) is widely used as a serum biomarker for predicting the clinical activity of inflammatory disorders, including IBD; however, the CRP levels are not always elevated in active IBD patients [6]. Currently, fecal calprotectin (fCal) has become widely used as an accurate biomarker for mucosal healing of UC [7]. However, the correlation of fCal with clinical symptoms is fair to poor in UC [8], and the usefulness of fCal in CD remains unclear due to the variable accuracy. Moreover, the fecal samples have problems, such as difficulty in performing on-demand sampling, instability of samples, and prolonged time until obtaining test results in many institutions. Furthermore, patients often forget to present stool samples during their hospital visits. Therefore, to develop treat-to-target strategies for IBD, serum biomarkers more accurately reflecting endoscopic disease activity are necessary.

Through comprehensive proteomics analysis of serum samples from rheumatoid arthritis patients treated with infliximab, we identified serum leucine-rich alpha-2 glycoprotein (LRG) as a novel biomarker of disease activity of rheumatoid arthritis [9]. LRG is a 50-kDa glycoprotein first reported by Haupt et al. in 1977 [10], containing eight leucine-rich repeat domains; however, its physiological function remains unclear. We previously reported that the serum LRG levels were correlated with the disease activity in both CD and UC patients [9, 11]. We have also demonstrated that the serum LRG levels were elevated in patients with active CD and UC with normal serum CRP levels [9, 11], and more strongly associated with mucosal healing of UC than the CRP level [12]. However, the correlation between serum LRG levels and endoscopic activity remains unknown. In addition, the usefulness of LRG in monitoring the disease activity of IBD during induction and maintenance therapy is unclear. Moreover, these studies are retrospective, and no prospective studies have been performed to investigate the clinical usefulness of LRG in predicting the clinical activity of IBD.

This prospective observational study (Predictor and biomarker: leucine-rich alpha-2 glycoprotein for inflammatory bowel disease treatment with adalimumab [PLANET]) aimed to investigate the association of serum LRG levels with disease activity and endoscopic activity in IBD patients treated with adalimumab for 52 weeks, thus examining the usefulness of LRG as a monitoring biomarker during induction and maintenance therapy.

## Methods

### Patients

This multicenter prospective observational study was conducted between January 2016 and March 2018 at 20 institutions throughout Japan. This study was registered with the University hospital Medical Information Network (UMIN000019958).

Patients initiated on adalimumab therapy were recruited and evaluated for 52 weeks or until treatment discontinuation. The inclusion criteria were as follows: age ≥ 15 years, having moderate-to-severe active IBD defined by a CD activity index (CDAI) score of ≥ 220 in CD or a Mayo score of ≥ 6 in UC [13], and displaying inadequate responses to conventional therapies including corticosteroids and immunomodulators. CD and UC were diagnosed on the basis of established diagnostic criteria [13]. Exclusion criteria included bowel resection within 12 weeks, patients with stoma or ileal pouch (excluding ileo-rectal anastomosis), prior exposure to adalimumab, history of primary non-response to infliximab or secondary non-response to infliximab at 10 mg/kg, and contraindications to adalimumab.

### Treatment

Adalimumab was administered at 160 mg subcutaneously at week 0, followed by 80 mg at week 2, and then 40 mg at every other week. In CD, the dose could be increased to 80 mg if response was lost.

The following drugs were permitted to be used concomitantly if the dosage was stabilized during the 4 weeks before the study and maintained until the end of the study. The dose of the following medications could be reduced in accordance with the patients’ condition; 5-aminosalysalicylic acid, azathioprine, 6-mercaptopurine, methotrexate, oral corticosteroids, antibiotics for CD, and enteral nutrition.

Concomitant use of biologics other than adalimumab, intravenous corticosteroids, rectal corticosteroids or 5-aminosalicylic acid, ciclosporin, tacrolimus, cytaphresis, and total parenteral nutrition was prohibited throughout the study.

### Study design

Patient visits were scheduled upon administration of the first dose of adalimumab (week 0), week 12, 24, and 52 after initiating adalimumab treatment. Serum LRG levels, fCal levels, CDAI scores in CD or Partial Mayo scores (PMS) in UC were evaluated at week 0, 12, 24, and 52. If a patient discontinued the study before week 52, these variables were assessed upon discontinuation. Serum LRG levels were measured with a NANOPIA^®^ LRG Kit based on the latex turbidimetry method (Sekisui Medical, Tokyo, Japan). Colonoscopy was performed at baseline (within 4 weeks prior to adalimumab initiation) and at week 12, and 52 for UC, and at baseline, week 24 and 52 for CD. Endoscopic activity was assessed as the Simple Endoscopic Score for Crohn's disease (SES-CD) in CD [14] or the Mayo endoscopic subscore (MES) in UC [15]. Endoscopic activity was scored by on-site investigators. Clinical remission was defined by a CDAI score of < 150 or a PMS of ≤ 2; Endoscopic remission was defined by a SES-CD of < 4 [16] or a MES of ≤ 1 [17].

The following variables were recorded: age, sex, height, weight, smoking, concomitant medication, history of biologics (drug name, period of use, and reason for discontinuation), disease duration, disease location based on Montreal classification criteria [18], history of bowel surgery (time and type of surgery), and the presence or absence of perianal lesions (fissuring ulcer, fistula, abscess, etc.).

### Endpoints

The primary endpoints of this exploratory study are the following: (1) changes in adalimumab treatment-related biomarkers and (2) the correlation between the biomarker levels and endoscopic activities.

### Statistical analysis

Continuous variables are presented as mean ± standard deviation (SD) values, and non-parametric ones as median with range. The Wilcoxon signed rank test was performed to compare non-parametric paired values, and the paired *t* test was performed to compare parametric paired values. Between-group differences were analyzed using the Kruskal–Wallis test for non-parametric data and Student’s *t* test for parametric values. Categorical values are presented as numbers (%) and differences were analyzed using the Chi square test or Fisher’s exact test. Pearson’s test was performed to analyze the association between biomarkers and activity indices. Values of *p* < 0.05 were considered statistically significant.

## Results

### Patients

A total of 81 patients (34 CD, 47 UC) were enrolled herein, and their background characteristics are summarized in Table 1. Eighty patients were enrolled as the full analysis set, and 56 patients (69.1%) completed 52 weeks of treatment (Supplementary Fig. 1).

**Table 1:** Baseline patient characteristics (*n* = 81)

Sex, female/male, *n* (%)	30 (37.0)/51 (63.0)
Age, years, median (range)	35.9 (15–70)
Body mass index, mean (SD)	20.1 (3.3)
Disease, CD/UC, *n* (%)	34 (42.0)/47 (58.0)
Disease duration, years, mean (SD)	5.9 (6.7)
Disease location in CD, ileitis/colitis/ileocolitis, *n* (%)	3 (8.8)/4 (11.8)/27 (79.4)
Disease location in UC, left-sided/extensive, *n* (%)	16 (34.0)/31 (66.0)
Perianal lesion in CD, *n* (%)	21 (61.8)
Concomitant medications	
5-aminosalycilic acid, *n* (%)	70 (86.4)
Thiopurine, *n* (%)	29 (35.8)
Corticosteroid, *n* (%)	43 (53.1)
Previous use of biologics, *n* (%)	8 (9.9)
History of bowel surgery in CD, *n* (%)	8 (23.5)
Current smoker, *n* (%)	3 (3.7)
Laboratory data	
LRG (µg/ml), *n* = 80, mean (SD)	27.4 (12.6)
CRP (mg/dl), *n* = 80, mean (SD)	1.69 (2.29)
fCal (µg/g), *n* = 71, mean (SD)	5867 (6766)
CDAI in CD, mean (SD)	271.5 (55.5)
SES-CD in CD, *n* = 33, mean (SD)	17.3 (7.5)
PMS in UC, mean (SD)	5.9 (1.3)
MES in UC, *n* = 45, mean (SD)	2.3 (0.6)

*SD* standard deviation, *LRG* leucine-rich alpha-2 glycoprotein, *CRP* C-reactive protein, *CDAI* Crohn’s disease activity index, *SES-CD* Simplified Endoscopic Score for Crohn’s disease, *PMS* partial Mayo score, *MES* Mayo endoscopic subscore

**Fig. 1:**
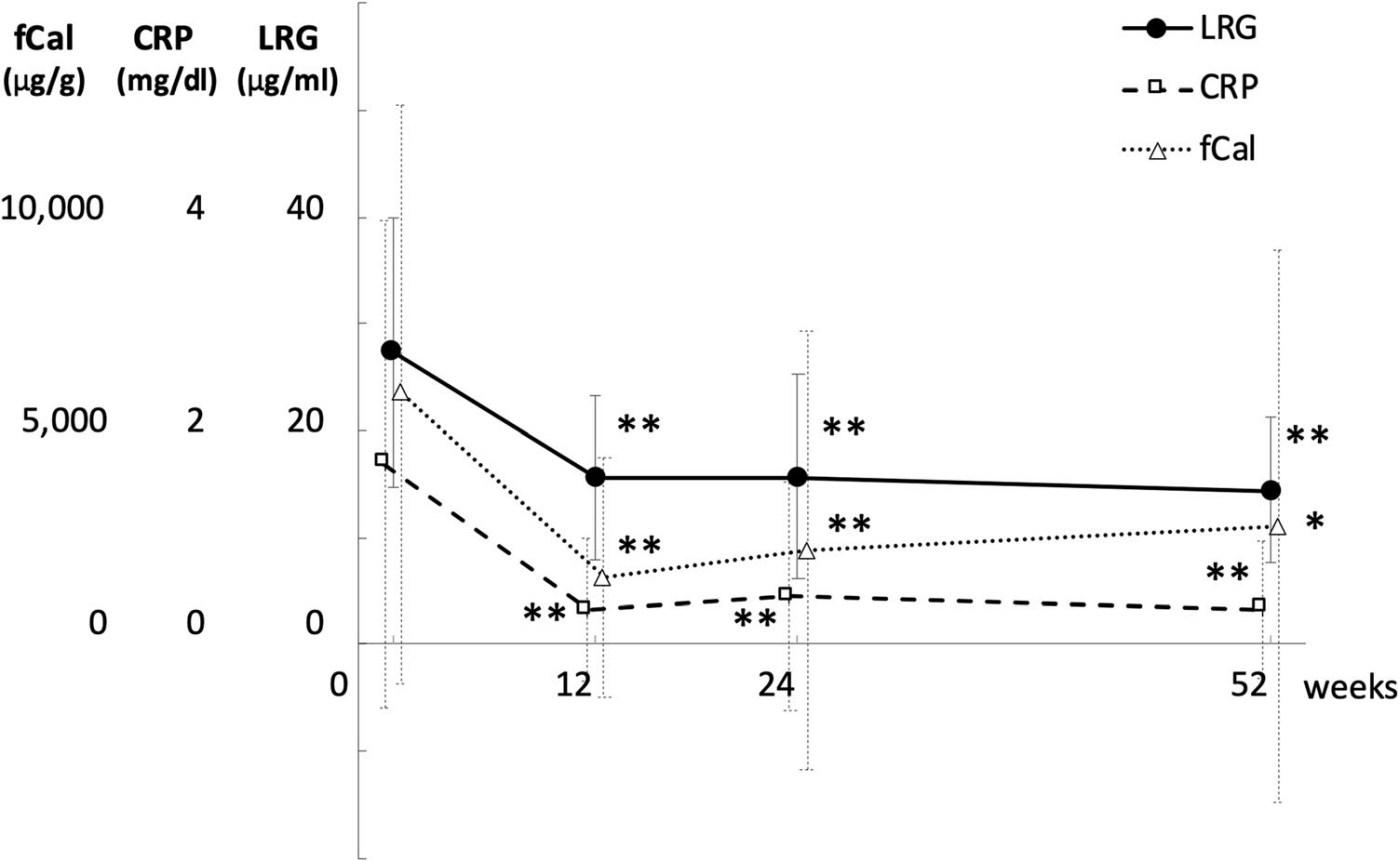
Changes in leucine-rich alpha-2 glycoprotein (LRG), C-reactive protein (CRP), and fecal calprotectin (fCal) during adalimumab treatment of IBD patients. Data were obtained before treatment (week 0) and at 12, 24, and 52 weeks after the treatment and shown in mean ± standard deviation (SD). **p < 0.001, *p < 0.01 vs. week 0 by paired *t* test

### Changes in disease activity upon initiation of adalimumab therapy

Given that this study was focused on the alteration of biomarkers by adalimumab treatment, we first analyzed the clinical and endoscopic efficacies among patients who continued adalimumab in each evaluation point. In total, 65.2% (45/69) of patients achieved clinical remission at week 12; week 24, 69.2% (45/65); week 52, 69.1% (38/55). Endoscopic remission was achieved in 44.2% (23/52) of patients at week 52 (Supplementary Fig. 2). In CD, both clinical and endoscopic activities significantly decreased upon initiation of adalimumab treatment (CDAI; 271.5 at week 0, 128.0 at week 12; *p* < 0.0001, 133.4 at week 24; *p* < 0.0001, and 115.9 at week 52; *p* < 0.0001 vs. week 0, SES-CD; 17.3 at week 0, 10.1 at week 24; *p* < 0.0001, and 8.6 at week 52; *p* < 0.0001 vs. week 0) (Supplementary Fig. 3a). Also, the clinical and endoscopic activities significantly decreased upon initiation of adalimumab treatment for UC (PMS; 5.9 at week 0, 2.0 at week 12; *p* < 0.0001, 1.4 at week 24; *p* < 0.0001, and 1.7 at week 52; *p* < 0.0001 vs. week 0, MES; 2.30 at week 0, 1.20 at week 12; *p* < 0.0001, and 1.2 at week 52; *p* < 0.0001 vs. week 0) (Supplementary Fig. 3b).

### Decreased serum LRG, CRP, and fCal levels with adalimumab treatment

Concurrent with clinical and endoscopic improvements, serum LRG levels significantly decreased from 27.4 ± 12.6 μg/ml at week 0 (*n* = 80) to 15.5 ± 7.7 μg/ml at week 12 (*n* = 70, *p* < 0.0001 vs. week 0), 15.7 ± 9.6 μg/ml at week 24 (*n* = 64, *p* < 0.0001 vs. week 0), and 14.5 ± 6.8 μg/ml at week 52 (*n* = 55, *p* < 0.0001 vs. week 0). Serum CRP levels also significantly decreased from 1.69 ± 2.29 mg/dl at week 0 to 0.33 ± 0.67 mg/dl at week 12 (*p* < 0.0001 vs. week 0), 0.45 ± 1.08 mg/dl at week 24 (*p* < 0.0001 vs. week 0), and 0.33 ± 0.65 mg/dl at week 52 (*p* < 0.0001 vs. week 0), and fCal levels significantly decreased from 5867 ± 6766 μg/g at week 0 to 1574 ± 2790 μg/g at week 12 (*p* < 0.0001), 2193 ± 5120 μg/g at week 24 (*p* = 0.0017 vs. week 0), and 2763 ± 6463 μg/g at week 52 (*p* = 0.036 vs. week 0) (Fig. 1). We also confirmed that the LRG, CRP and fCal levels were significantly decreased at 12, 24, and 52 weeks when the patients were divided into CD and UC (Supplementary Fig. 4).

### LRG, CRP, and fCal levels at each time point in patients with and without endoscopic remission at week 52

**Fig. 2:**
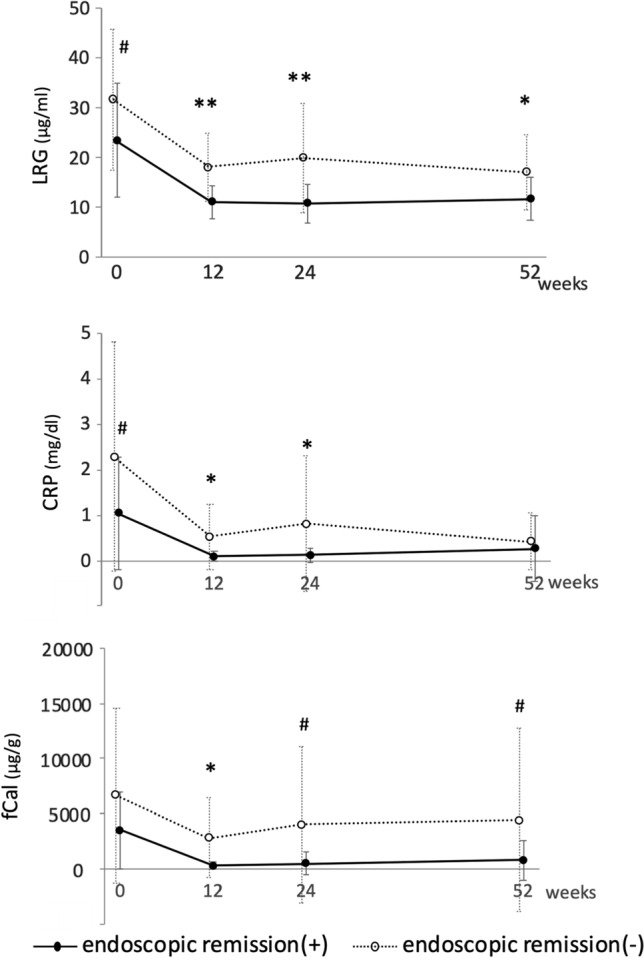
Changes in the biomarkers in IBD patients before and after the adalimumab treatment depending on the response to treatment. Levels of LRG, CRP, and fCal in patients with endoscopic remission at week 52 (*n* = 23, solid line) and those without (*n* = 29, dotted line) were shown in mean ± SD and compared in each time point. **p < 0.001, *p < 0.01, # *p* < 0.05 by Student’s *t* test

LRG, CRP, and fCal levels at each time point were stratified by endoscopic remission at week 52 (Fig. 2). The mean LRG levels at week 0. 12, 24, and 52 were significantly lower in patients with endoscopic remission at week 52 compared to those who did not (week 0; 23.4 μg/ml vs. 31.6 μg/ml; *p* = 0.0244, week 12; 11.2 μg/ml vs. 18.1 μg/ml; *p* < 0.0001, week 24; 10.8 μg/ml vs. 20.0 μg/ml; *p* = 0.0002, week 52; 11.7 μg/ml vs. 17.1 μg/ml; *p* = 0.026). The mean CRP level was significantly lower in patients with endoscopic remission than those who did not at week 0, 12, 24, but not at week 52 (week 0; 1.04 mg/dl vs. 2.28 mg/dl; *p* = 0.0251, week 12; 0.10 mg/dl vs. 0.53 mg/dl; *p* = 0.0033, week 24; 0.12 mg/dl vs. 0.82 mg/dl; *p* = 0.0175, week 52; 0.26 mg/dl vs. 0.42 mg/dl; *p* = 0.3941). Also, the mean fCal level was significantly lower in patients with endoscopic remission than those who did not at week 12, 24, 52, but not at week 0 (week 0; 3492 μg/g vs. 6632 μg/g; *p* = 0.0767, week 12; 267 μg/g vs. 2798 μg/g; *p* = 0.0011, week 24; 481 μg/g vs. 4024 μg/g; *p* = 0.0124, week 52; 796 μg/g vs. 4433 μg/g; *p* = 0.3941).

### Correlation between disease activity and biomarkers

We assessed the correlation between disease activity and biomarker levels at each time point (Table 2). In CD, LRG levels were correlated with CDAI scores at weeks 12, 24 and 52. Furthermore, CRP levels were correlated with CDAI scores at weeks 0, 24, and 52, while fCal levels were significantly correlated with CDAI scores only at week 52. In UC, LRG levels were also correlated with PMS scores at each time point. CRP levels were correlated with PMS scores at weeks 0, 12, and 24, while fCal levels were significantly correlated with PMS scores only at week 12 and 52.

**Table 2:** Correlation of serum and fecal biomarkers with clinical disease activity

		Week 0			Week 12			Week 24			Week 52		
		*n*	*r*	*p*	*n*	*r*	*p*	*n*	*r*	*p*	*n*	*r*	*p*
CDAI (CD)	LRG	34	0.233	0.1851	33	0.440	0.0105	33	0.743	< 0.0001	28	0.554	0.0022
	CRP	34	0.375	0.0287	33	0.339	0.0540	33	0.622	0.0001	28	0.597	0.0008
	fCal	32	−0.097	0.5982	31	0.014	0.9387	32	0.204	0.2616	27	0.600	0.0009
PMS (UC)	LRG	46	0.503	0.0004	36	0.402	0.0151	31	0.376	0.0368	27	0.398	0.0398
	CRP	46	0.440	0.0022	35	0.372	0.0280	32	0.441	0.0115	27	0.072	0.7204
	fCal	39	0.108	0.5117	36	0.411	0.0127	31	0.327	0.0730	24	0.494	0.0141

*CDAI* Crohn’s disease activity index, *PMS* partial Mayo score, *LRG* leucine-rich alpha-2 glycoprotein, *CRP* C-reactive protein, *fCal* fecal calprotectin

### Correlation between endoscopic activity and biomarkers

We next evaluated the correlation between endoscopic activity and biomarker levels at each time point (Table 3). In CD, LRG levels were correlated with SES-CD at each time point, while CRP levels were correlated with SES-CD only at week 24 and fCal levels at weeks 24 and 52. In UC, LRG levels were also correlated with MES at weeks 0 and 12, while CRP levels were correlated with MES only at week 0 and fCal levels at week 12.

**Table 3:** Correlation of serum and fecal biomarkers with endoscopic disease activity

		Week 0			Week 12			Week 24			Week 52		
		*n*	*r*	*p*	*n*	*r*	*p*	*n*	*r*	*p*	*n*	*r*	*p*
SES-CD (CD)	LRG	33	0.391	0.0244	–	–	–	27	0.563	0.0022	25	0.697	0.0001
	CRP	33	0.107	0.5536	–	–	–	27	0.470	0.0134	25	0.384	0.0582
	fCal	31	0.300	0.1005	–	–	–	26	0.659	0.0003	25	0.593	0.0398
MES (UC)	LRG	45	0.534	0.0002	31	0.429	0.0159	–	–	–	27	0.335	0.0877
	CRP	45	0.538	0.0001	30	0.234	0.2137	–	–	–	27	− 0.022	0.9134
	fCal	38	0.194	0.2430	31	0.448	0.0116	–	–	–	24	0.393	0.0578

*SES-CD* Simplified Endoscopic Score for Crohn's disease, *MES* Mayo endoscopic subscore, *LRG* leucine-rich alpha-2-glycoprotein, *CRP* C-reactive protein, *fCal* fecal calprotectin

**Fig. 3:**
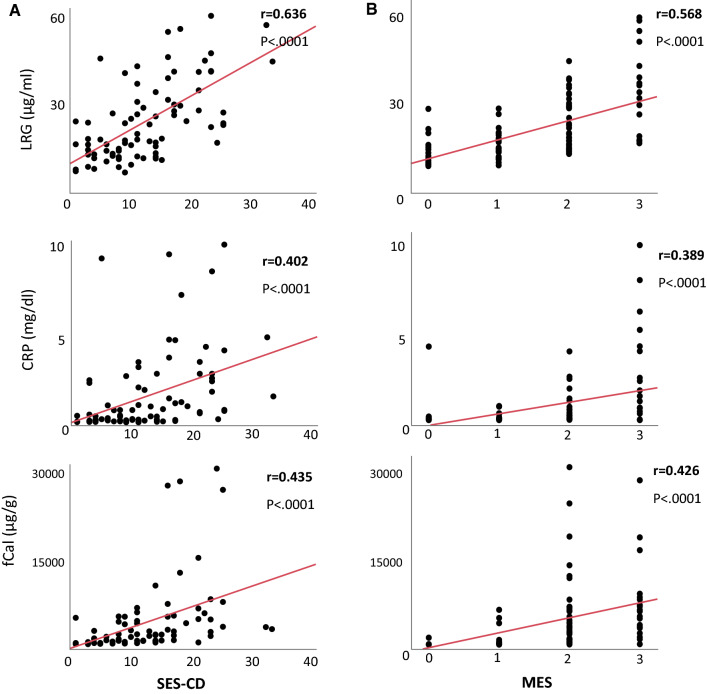
Correlation of serum and fecal biomarkers with endoscopic activities. All serial time points were put into an analysis, and the correlation of biomarkers with (**a**) simple endoscopic score for Crohn’s disease (SES-CD) in CD or (**b**) Mayo endoscopic subscore (MES) in UC are shown

Each biomarker was assessed at all time points together to evaluate the correlation of each biomarker with clinical activity (Supplementary Fig. 5) and endoscopic activity (Fig. 3). Among the three biomarkers, LRG was most significantly correlated with both clinical activity indices (CDAI and PMS) and endoscopic activity indices (SES-CD and MES).

## Discussion

Herein, we assessed LRG, CRP, fCal and disease activity indices serially during a 52-week period among IBD patients treated with adalimumab for induction and maintenance of remission. LRG levels reflected clinical and endoscopic disease activity at each predetermined time point. The correlation between LRG and disease activity was superior to that of CRP and fCal at each time point. LRG levels better correlated with endoscopic activity than CRP and fCal, indicating that LRG is a potential surrogate marker for endoscopic activity and useful for monitoring disease activity during the induction and maintenance of remission in IBD.

Ideal biomarkers are simple, easy to screen, noninvasive, cost-effective, rapid, and reproducible [6]. Considering these biomarker requirements, LRG has several advantages over other preclinical serum biomarkers reportedly used to assess IBD disease activity [19, 20]. A commercial LRG assay has been approved and is available for medical use in Japan, yielding results in 10 min. Thus, LRG can be assessed in daily clinical practice.

The treat-to-target strategy has been introduced for IBD treatment. The CALM trial assessed the effects of this strategy in CD [21]. This study shows that additional assessment of CRP and fCal along with symptoms to facilitate decisions regarding treatment modifications improves endoscopic remission rates at 52 weeks after adalimumab initiation. These results show the importance of monitoring disease activity during induction therapy through objective analysis. Thus far, CRP and fCal have been assessed as objective disease activity biomarkers for IBD during the clinical management of IBD [4]. However, serum CRP levels may not be elevated even in patients with active mucosal inflammation [22]. Although fCal is correlated with mucosal inflammatory activity in UC [23], limited information is available regarding whether it is correlated with disease activity during small intestinal mucosal inflammation in CD [24–26]. Furthermore, stool sampling is troublesome for patients. Therefore, serum biomarkers more accurately reflecting the endoscopic activity of IBD are warranted.

Herein, LRG displayed a higher correlation with endoscopic activity than CRP, probably because CRP is produced by hepatocytes solely in response to interleukin (IL)-6 stimulation, whereas LRG is induced not only by IL-6 but also by several other proinflammatory cytokines including IL-22 and TNF [11]. Furthermore, LRG is produced by intestinal epithelial cells [11], thus potentially reflecting inflammatory activity more sensitively.

We also clarified that LRG was significantly lower in patients with endoscopic remission at week 52 than those without at all time points, but CRP and fCal showed no differences at week 52 and week 0, respectively (Fig. 2). This indicates that LRG can stably predict the long-term endoscopic response at week 52 during the adalimumab treatment course. Given that TNF is one of the cytokines that drive LRG production [11], theoretically, anti-TNF treatment can influence the serum LRG levels. However, our results clearly showed that LRG was strongly associated with the disease activity indices, even during the treatment with adalimumab. These results suggest that LRG can be useful for monitoring the disease activity, even after the anti-TNF therapy.

The present results support the use of LRG during induction therapy for IBD as follows: LRG levels can be determined before treatment initiation for induction of remission and then measured periodically to monitor changes in disease activity. If LRG levels do not decrease, treatment should be intensified. If LRG levels are within normal levels, colonoscopy should be performed to confirm mucosal healing, which is currently the most reliable therapeutic goal, and maintenance therapy can be continued. The cutoff value of LRG, like fCal, can vary depending on the disease location and clinical manifestation, and further investigation is required.

This study has several limitations. First, we are unsure whether the present results may be generalized to IBD therapies other than adalimumab, because we only enrolled patients receiving adalimumab therapy. Second, histological analysis has not been performed in the present study, because our aim was to consolidate the usefulness of LRG in monitoring mucosal healing prospectively. Third, it was unclear whether the presence of extra-intestinal manifestations, perianal fistula and deep small bowel lesions affected LRG levels. A larger study, including patients receiving various induction therapies and subjected to another activity indices including histological analysis, is needed to clarify these issues.

In conclusion, this study shows that LRG, a novel serum biomarker, better reflects endoscopic activity during adalimumab treatment than CRP or fCal among IBD patients, indicating that LRG can be considered a potential biomarker to monitor disease activity during induction and maintenance therapy and can be incorporated in the treat-to-target strategy for the clinical management of IBD.

## Supplementary Information

Below is the link to the electronic supplementary material.

Supplementary file1 (PPTX 2776 KB)

## Data Availability

The data underlying this article will be shared on reasonable request to the corresponding author.
