# Supplementary material for: Leucine-rich alpha-2 glycoprotein is a potential biomarker to monitor disease activity in inflammatory bowel disease receiving adalimumab: PLANET study
Source: J Gastroenterol. 2021 May 3;56(6):560–9. doi: 10.1007/s00535-021-01793-0 (PMC8137624; doi:10.1007/s00535-021-01793-0)
Supplement: Supplementary file 1 — Supplementary file1 (PPTX 2776 KB) [file 535_2021_1793_MOESM1_ESM.pptx]

## Slide 1
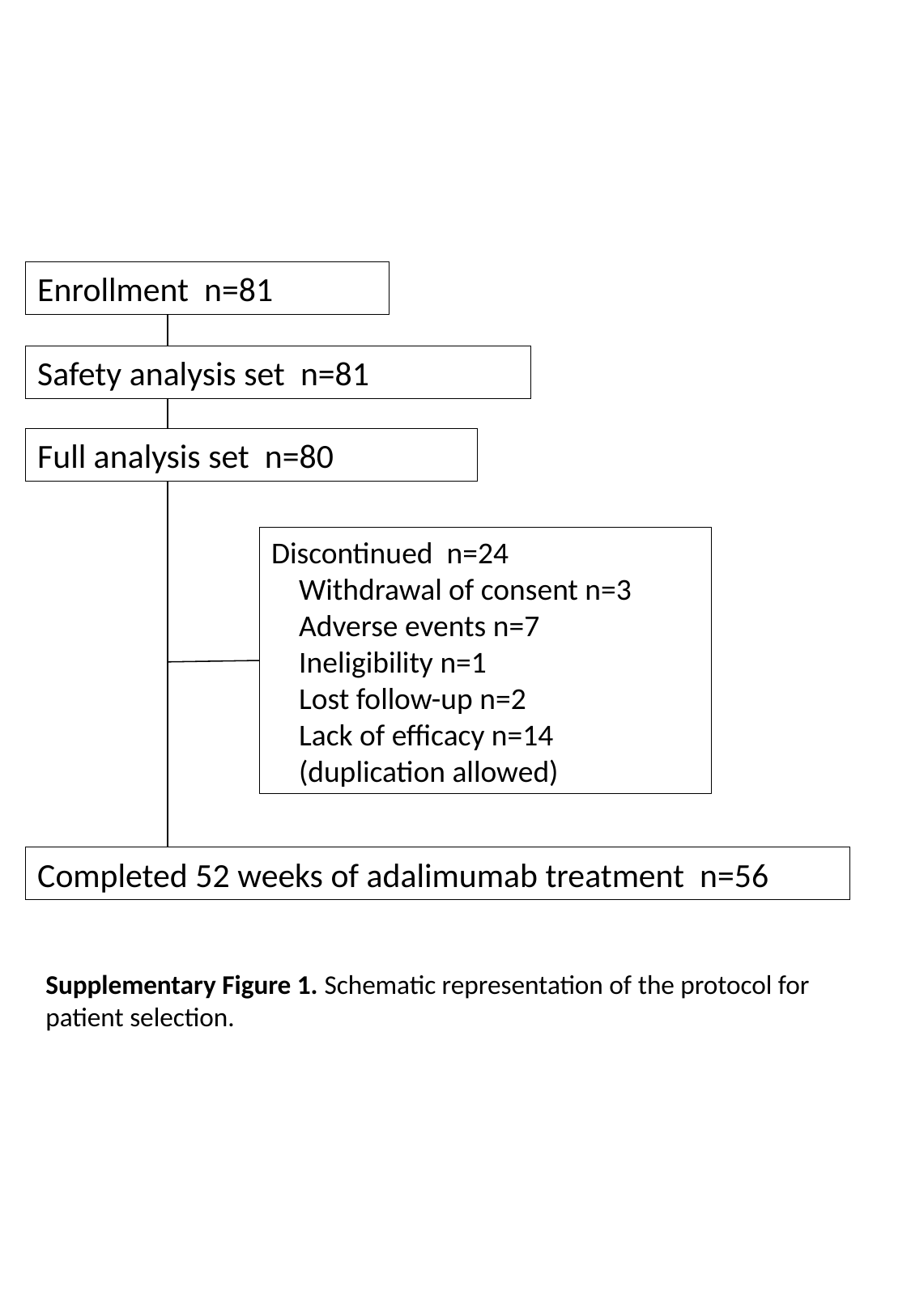

Enrollment n=81
Safety analysis set n=81
Full analysis set n=80
Discontinued n=24
 Withdrawal of consent n=3
 Adverse events n=7
 Ineligibility n=1
 Lost follow-up n=2
 Lack of efficacy n=14
 (duplication allowed)
Completed 52 weeks of adalimumab treatment n=56
Supplementary Figure 1. Schematic representation of the protocol for patient selection.

## Slide 2
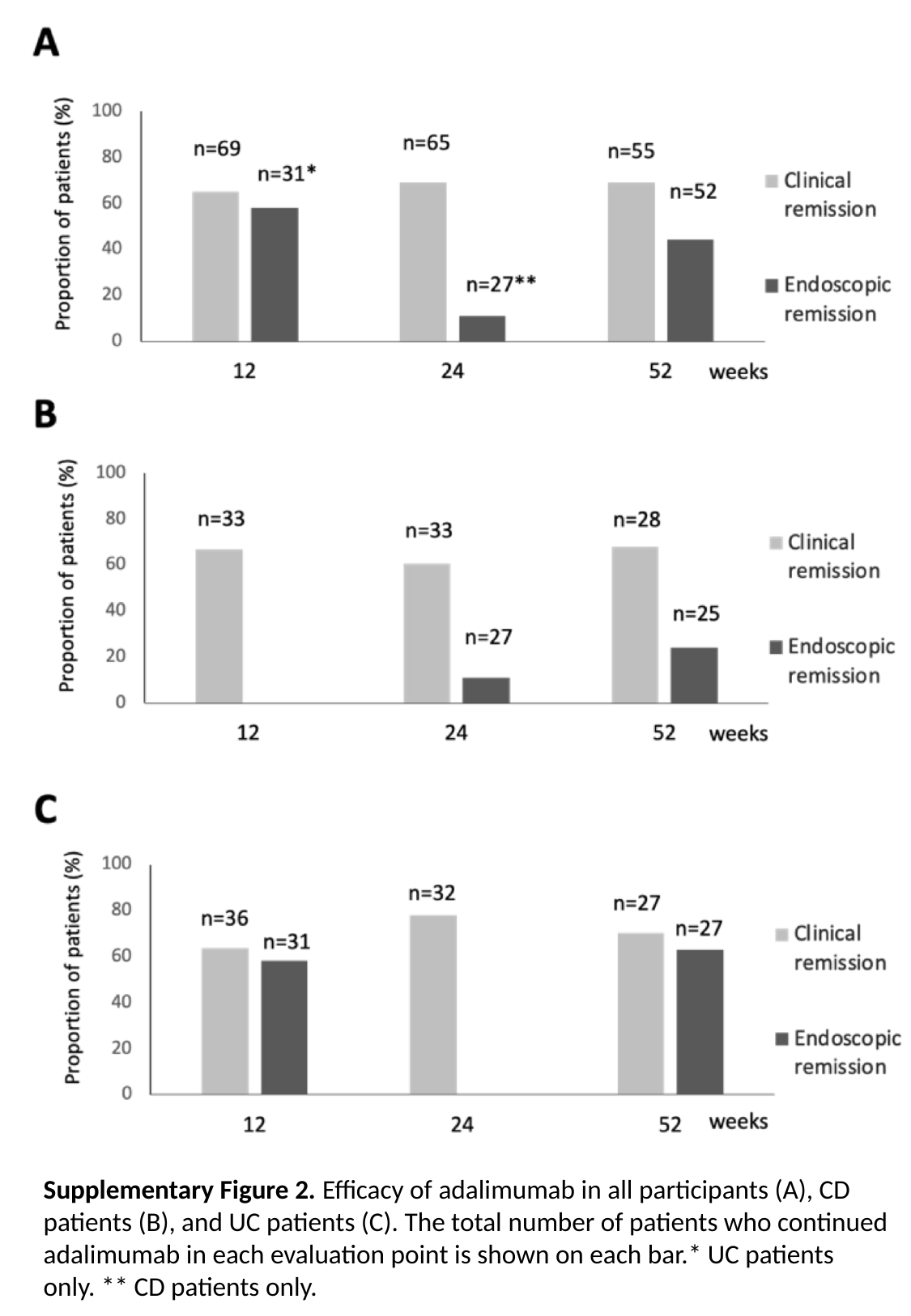

Supplementary Figure 2. Efficacy of adalimumab in all participants (A), CD patients (B), and UC patients (C). The total number of patients who continued adalimumab in each evaluation point is shown on each bar.* UC patients only. ** CD patients only.

## Slide 3
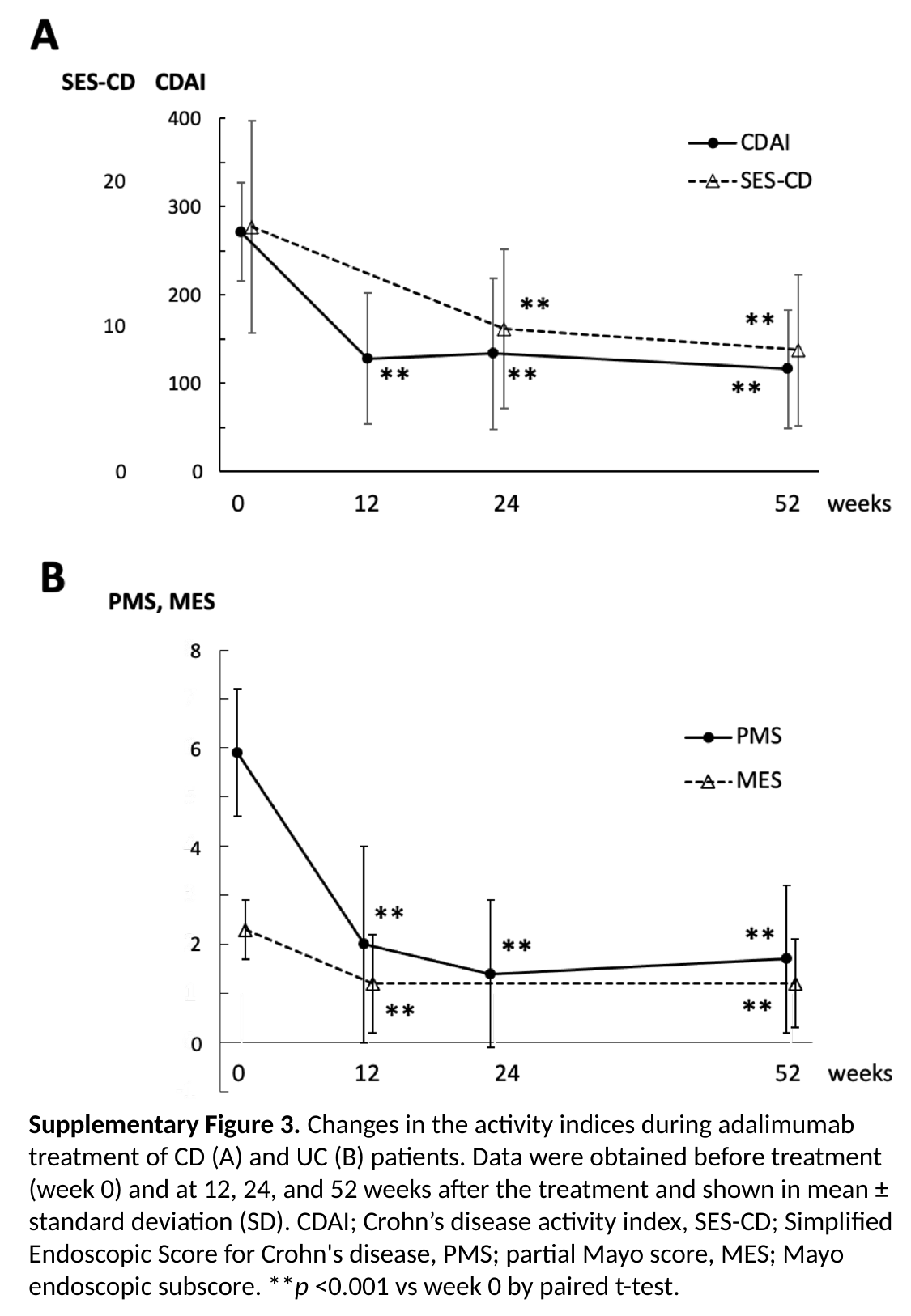

Supplementary Figure 3. Changes in the activity indices during adalimumab treatment of CD (A) and UC (B) patients. Data were obtained before treatment (week 0) and at 12, 24, and 52 weeks after the treatment and shown in mean ± standard deviation (SD). CDAI; Crohn’s disease activity index, SES-CD; Simplified Endoscopic Score for Crohn's disease, PMS; partial Mayo score, MES; Mayo endoscopic subscore. **p <0.001 vs week 0 by paired t-test.

## Slide 4
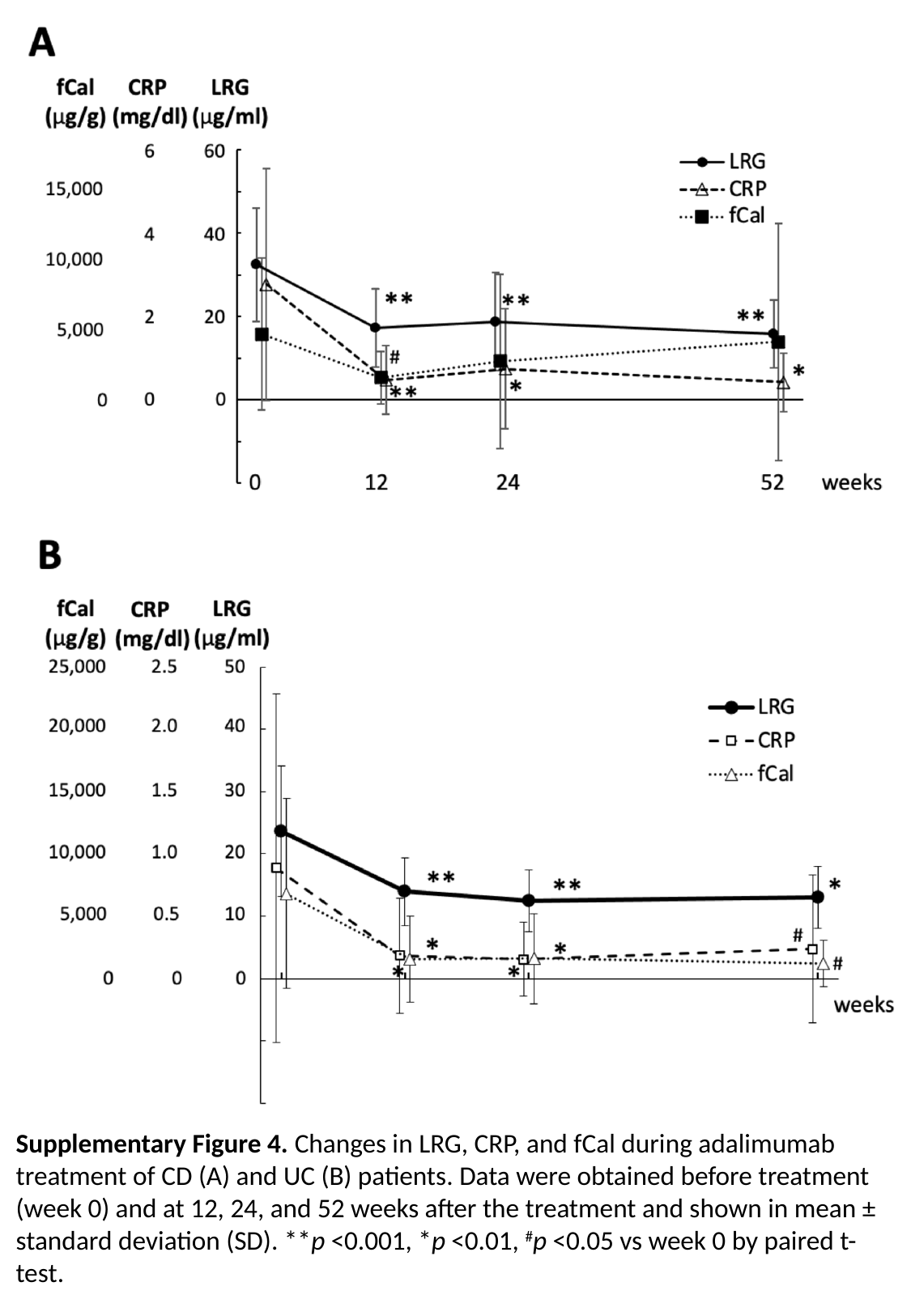

Supplementary Figure 4. Changes in LRG, CRP, and fCal during adalimumab treatment of CD (A) and UC (B) patients. Data were obtained before treatment (week 0) and at 12, 24, and 52 weeks after the treatment and shown in mean ± standard deviation (SD). **p <0.001, *p <0.01, #p <0.05 vs week 0 by paired t-test.

## Slide 5
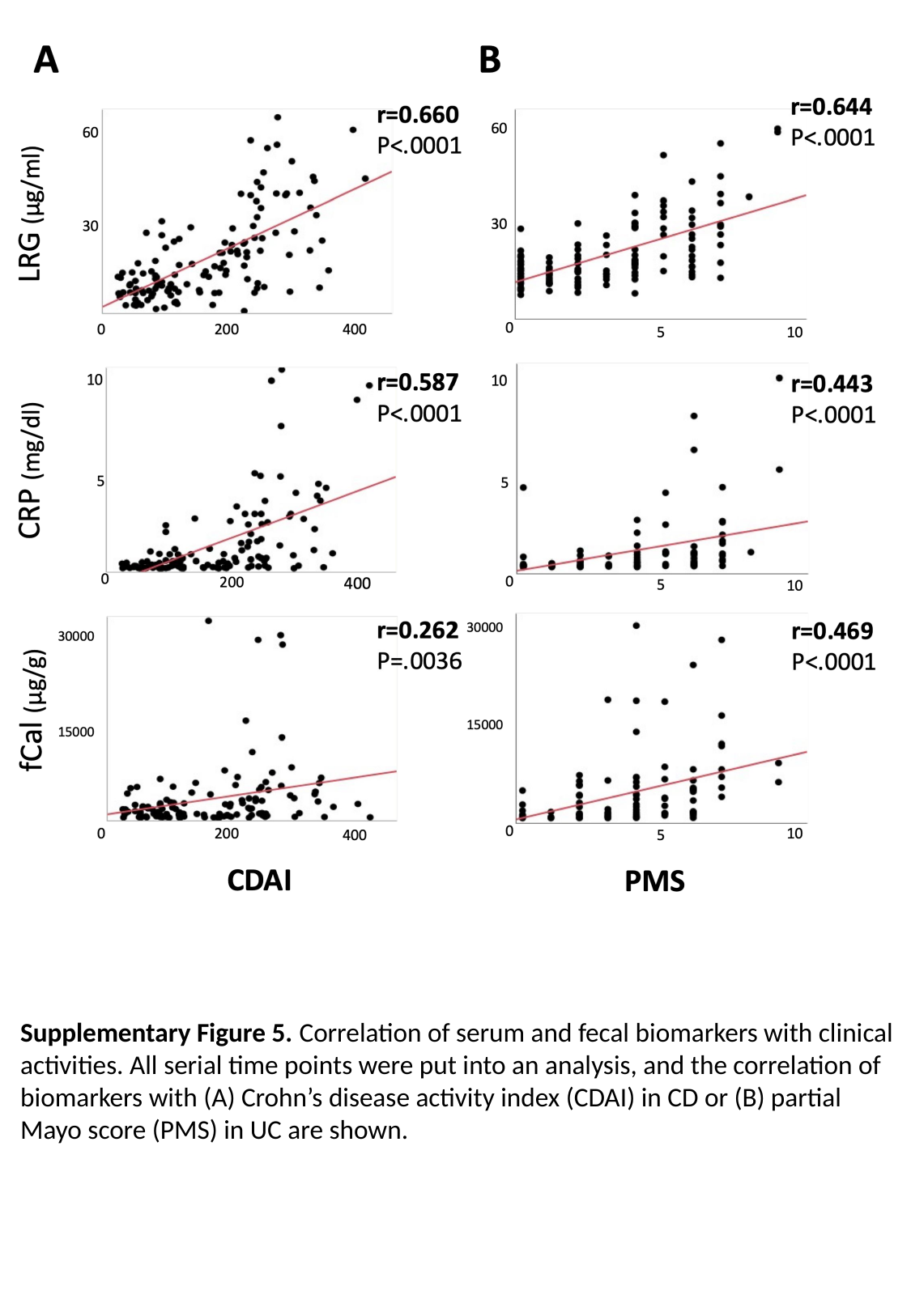

Supplementary Figure 5. Correlation of serum and fecal biomarkers with clinical activities. All serial time points were put into an analysis, and the correlation of biomarkers with (A) Crohn’s disease activity index (CDAI) in CD or (B) partial Mayo score (PMS) in UC are shown.
